# Catestatin attenuates endoplasmic reticulum induced cell apoptosis by activation type 2 muscarinic acetylcholine receptor in cardiac ischemia/reperfusion

**DOI:** 10.1038/srep16590

**Published:** 2015-11-16

**Authors:** Feng Liao, Yang Zheng, Junyan Cai, Jinghui Fan, Jing Wang, Jichun Yang, Qinghua Cui, Guoheng Xu, Chaoshu Tang, Bin Geng

**Affiliations:** 1 Department of Physiology and Pathophysiology, School of Basic Medical Science, Peking University, P.R. China; 2 Center for Noncoding RNA Medicine, Peking University Health Science Center Beijing 100191, China.

## Abstract

Catestatin (CST) is a catecholamine secretion inhibiting peptide as non-competitive inhibitor of nicotinic acetylcholine receptor. CST play a protective role in cardiac ischemia/reperfusion (I/R) but the molecular mechanism remains unclear. Cardiomyocytes endogenously produced CST and its expression was reduced after I/R. CST pretreatment decreased apoptosis especially endoplasmic reticulum (ER) stress response during I/R. The protection of CST was confirmed in H9c2 cardiomyoblasts under Anoxia/reoxygenation (A/R). In contrast, siRNA-mediated knockdown of CST exaggerated ER stress induced apoptosis. The protective effects of CST were blocked by extracellular signal-regulated kinases 1/2 (ERK1/2) inhibitor PD90895 and phosphoinositide 3-kinase (PI3 K) inhibitor wortmannin. CST also increased ERK1/2 and protein kinase B (Akt) phosphorylation and which was blocked by atropine and selective type 2 muscarinic acetylcholine (M2) receptor, but not type 1 muscarinic acetylcholine (M1) receptor antagonist. Receptor binding assay revealed that CST competitively bound to the M2 receptor with a 50% inhibitory concentration of 25.7 nM. Accordingly, CST inhibited cellular cAMP stimulated by isoproterenol or forskolin, and which was blocked by selective M2 receptor antagonist. Our findings revealed that CST binds to M2 receptor, then activates ERK1/2 and PI3 K/Akt pathway to inhibit ER stress-induced cell apoptosis resulting in attenuation cardiac I/R injury.

Catestatin (CST) is a 21 amino acid-residue, hydrophobic neuroendocrine peptide derived from chromogranin A (ChgA)^1^. It is co-stored in the secretory granule and co-released with catecholamine in adrenal chromaffin cells and adrenergic neurons as an endogenous noncompetitive antagonist of nicotine acetylcholine (nAch) receptor and inhibition catecholamine secretion in mammals^2^. CST stimulated histamine release from mast cells^3^,^4^. CST also regulated rostral ventrolateral medulla neuron activity and caused reduction sympathetic barosensitivity and parapheral chemoreflex^5^,^6^. Clearly, CST has a significant anti-hypertensive effect^7^.

CST is also expressed and generated in the heart^8^. CST reduces isoproterenol and endothelin-1–induced cardiac contractility^9^,^10^ through the PI3 K-Akt-endothelial nitric oxide synthase (eNOS) pathway^11^. Many clinical trials have revealed an association between plasma CST level and cardiac diseases including acute myocardial infarction, heart failure and cardiac remodeling^12^,^13^,^14^,^15^. These studies suggest that CST may play an essential role in the pathogenesis of ischemic heart diseases. More recently, Penna *et al*. found that CST post-conditioning or post-treatment decreased infarct area and diastolic left ventricular (LV) pressure, and increased cardiomyocyte viability during cardiac ischemic-reperfusion (I/R)^16^. PI3 K and protein kinase Cε inhibitors, mitochondrial K_ATP_ channel blocker and reactive oxygen species (ROS) scavenger abolished the protective effects of CST in cardiac I/R^17^. Clearly, to investigate the protective mechanism of endogenous CST in cardiac diseases will be very useful and necessary for its potential clinical use.

It had been reported that activation of nAch (α7nAchR)^18^ or type 2 muscarinic Ach receptor (M2 receptor)^19^ reduced cardiac I/R injury. Because CST is a noncompetitive inhibitor of nAch receptor^1^, it is likely that nAch receptor is not involved in protective effect of CST. Interestingly, Gi/o protein participated in the cardiac negative inotropism of CST^10^,^20^. Gi/o protein is also an important intracellular target of M2 and M4 receptors^21^. Based on these previous findings, it is reasonably to speculate that M receptor may play essential roles in CST’s protective effects during cardiac I/R injury.

In the present study, we presented novel evidence that CST directly bound to and activated M2 receptor to attenuate ER-stress linked apoptosis via activation of ERK1/2 and Akt pathways in cardiac I/R.

## Results

### CST pretreatment improved left ventricular (LV) function and decreased I/R injury

**Figure 1:**
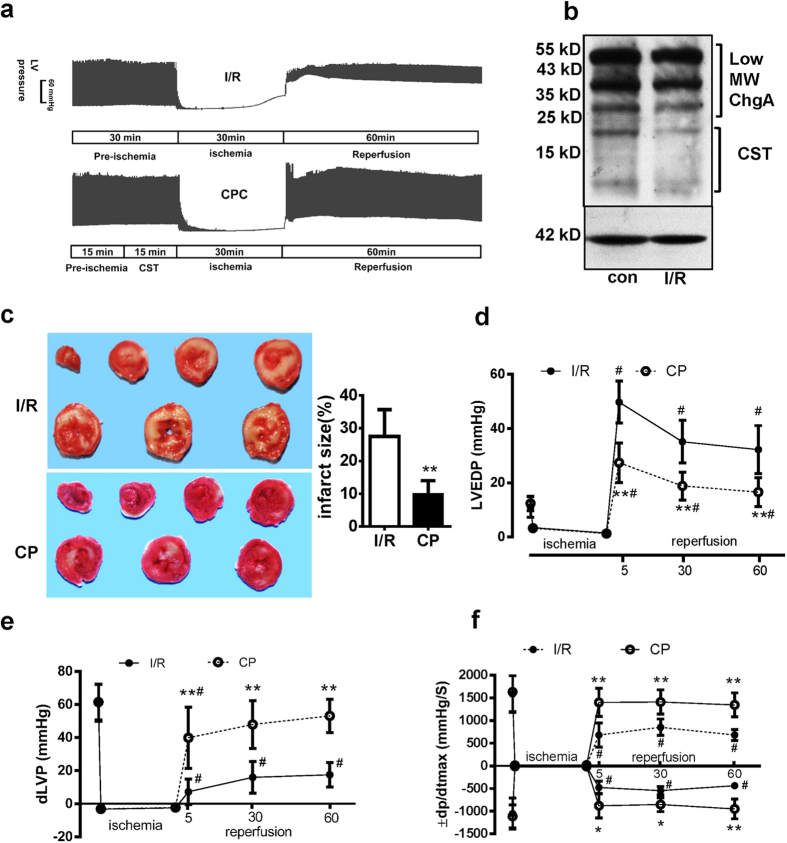
Catestatin (CST) pretreatment (CP) reduced infarct size and maintained cardiac function after cardiac ischemia-reperfusion (I/R) injury in rats. (**a**) The procedure of global cardiac I/R (n = 18) and CP (n = 18) and original recording of left ventricular (LV) pressure. (**b**) The ChgA protein and its cleavage fragments expression were measured by western blot. (**c**) Heart tissue was stained with triphenyltetrazolium chloride and infarct size (white color) was measured by volume (n = 6 for each group). Evaluation of cardiac function including LV end diastolic pressure (LVEDP)(**b**), LV developed pressure (dLVP, LV systolic pressure–diastolic pressure) (**d**), and LV ±dp/dt_max_ (**e**) during I/R and CP (total 36 rats were recruited). Data are mean ± SD. *P < 0.05, **P < 0.01 vs I/R group. ^#^P < 0.05 vs parameters before ischemia.

Ischemia reperfusion procedure and CST pretreatment (CP) (CST supplementation for 15 min before I/R) were performed (Fig. 1a). After 30 min of ischemia and 1 h of reperfusion, cardiac endogenous ChgA mRNA (see supplementary Fig. S1 online) and protein expression were no changes, but CST fragments (21 kD and 7.5 kD) reduced comparison to control (Fig. 1b). Different dose of CST perfusion for 1 h did not induce lactate dehydrogenase (LDH) leakage (supplementary Fig. S2 online). CST (25 nM, 50 nM and 100 nM) pretreatment decreased LDH leakage and cardiac troponin I (cTNI) level in perfusate at different reperfusion time point (supplementary Fig. S3 online), which indicated that CST pretreatment lowered the ischemia reperfusion (I/R) injury, especially CST at 100 nM exhibited well protection. So we selected 100 nM CST in following experiments. As Fig. 1c showed, I/R caused global cardiac infarction, and CP significantly reduced infarct size as compared with I/R alone (9.8 ± 4.2% vs 27.5 ± 8.2%, *P* = 0.0001).

We also assessed LV diastolic pressure (LVEDP), developed LV pressure (LV systolic pressure minus LVEDP, dLVP) and maximal rate of LV pressure development (LV ± dp/dtmax) for LV function. The I/R rat heart showed greatly increased LVEDP and impaired dilation; CP lowered the LVEDP during each time point of reperfusion (All *P* < 0.05, Fig. 1d). CP also improved the dLVP recovery (All *P* < 0.05, Fig. 1e) and LV ± dp/dtmax (All *P* < 0.05 Fig. 1f). Thus, CST pretreating improved cardiac function during I/R.

### CST pretreatment inhibited I/R–induced cell apoptosis

**Figure 2:**
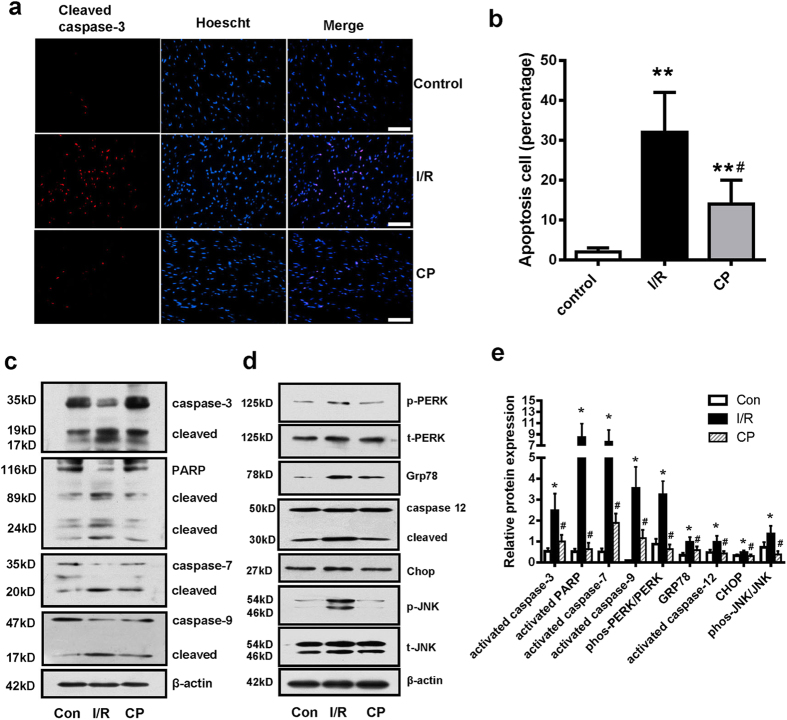
CST pretreatment reduced ER-stress and decreased apoptosis. (**a**) Cleaved caspase-3 immunofluorescence staining and nuclear staining; apoptotic cells were stained in pink Scale bar = 100 μM. (**b**) Apoptosis cell numbers were counted in five different field of vision. (**c**) Western blot analysis of effect of I/R and CP on apoptosis and caspase family members and PARP and (**d**) proteins related to the ER stress pathway. Relative protein expression was analyzed by band gray degree. β-actin is a normalization control (**e**). Six independent experiments were performed for above studies.

We used cleaved caspase-3 (a marker of cell apoptosis) antibody immunofluorescence staining to assess cell apoptosis. I/R increased the number of apoptotic cardiomyocytes, and CP significantly reduced the cell apoptosis (Fig. 2a,b). Consistently, I/R increased apoptosis markers (caspase-9, 7 and -3 cleavage, and poly (ADP-ribose) polymerase (PARP) cleaved into 89- and 24-kDa segments). CP lowered cleaved caspase-9, -7, -3 and PARP (Fig. 2c,e) for a role in reduced apoptosis.

I/R induced ER-stress response by assessing phosphorylated PERK and chaperone protein Grp78 expression. ER stress-initiated apoptosis signals evaluated by caspase-12 cleavage, Chop protein expression and phosphorylated JNK. CP also lowered the protein expression of ER stress markers and ER stress-initiated apoptosis signals in cardiac I/R (Fig. 2d,e).

**Figure 3:**
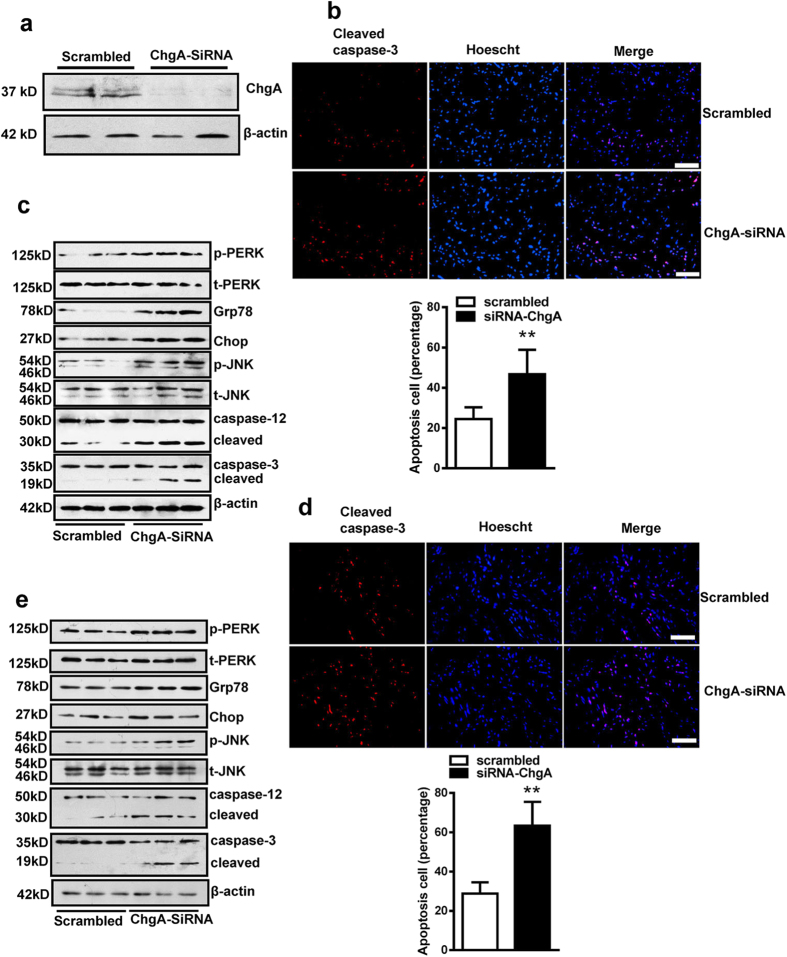
siRNA knockdown of CST increased ER stress-induced apoptosis in H9c2 cardiomyoblasts during anoxia/reperfusion (A/R). (**a**) siRNA knockdown effect by CST precursor (ChgA) protein expression. Immunofluorescence staining by cleaved caspase-3 for cell apoptosis (**b**) and western blot analysis of ER stress-marker expression (**c**). Cells with CST siRNA knockdown were treated with thapsigargin; immunohistochemistry of apoptosis and (**d**) western blot analysis of ER stress-markers expression (**e**). Five independent experiments were performed for these studies. Scale bar = 100 μM.

To confirm the CST regulatory role in ER stress-induced apoptosis, we used siRNA knockdown of the CST precursor ChgA in H9c2 cardiomyoblasts. Firstly, we measured the cell viability by CCK8 kit and found that different CST treatment or knockdown did not affect H9c2 cell viability (see supplementary Fig. S4a,S4b online). siRNA treatment for 48 h lowered CST precursor-ChgA protein expression in H9c2 cells (Fig. 3a). In H9c2 cell with A/R, siRNA mediated knockdown of ChgA increased the number of apoptotic cells as compared with scramble siRNA (Fig. 3b). ChgA siRNA also upregulated ER stress response markers (phosphorylated PERK and JNK, Grp78 and Chop expression, and cleaved caspase-12) and an apoptosis marker (cleaved caspase-3) (Fig. 3c). Calcium overload is an important injury mechanism in I/R and an inducer of ER stress. Thapsigargin (calcium pump inhibitor induce intracellular calcium overload) is a typical ER stress inducer. Knockdown of CST also increased thapsigargin-induced cell apoptosis (Fig. 3d) and especially levels of the ER stress markers and cell apoptosis marker (Fig. 3e and supplementary Fig. S5 online). Above results suggest that endogenous CST may reduce ER stress in part with I/R injury.

### ERK and PI3 K pathway is involved in the protective effect of CST

**Figure 4:**
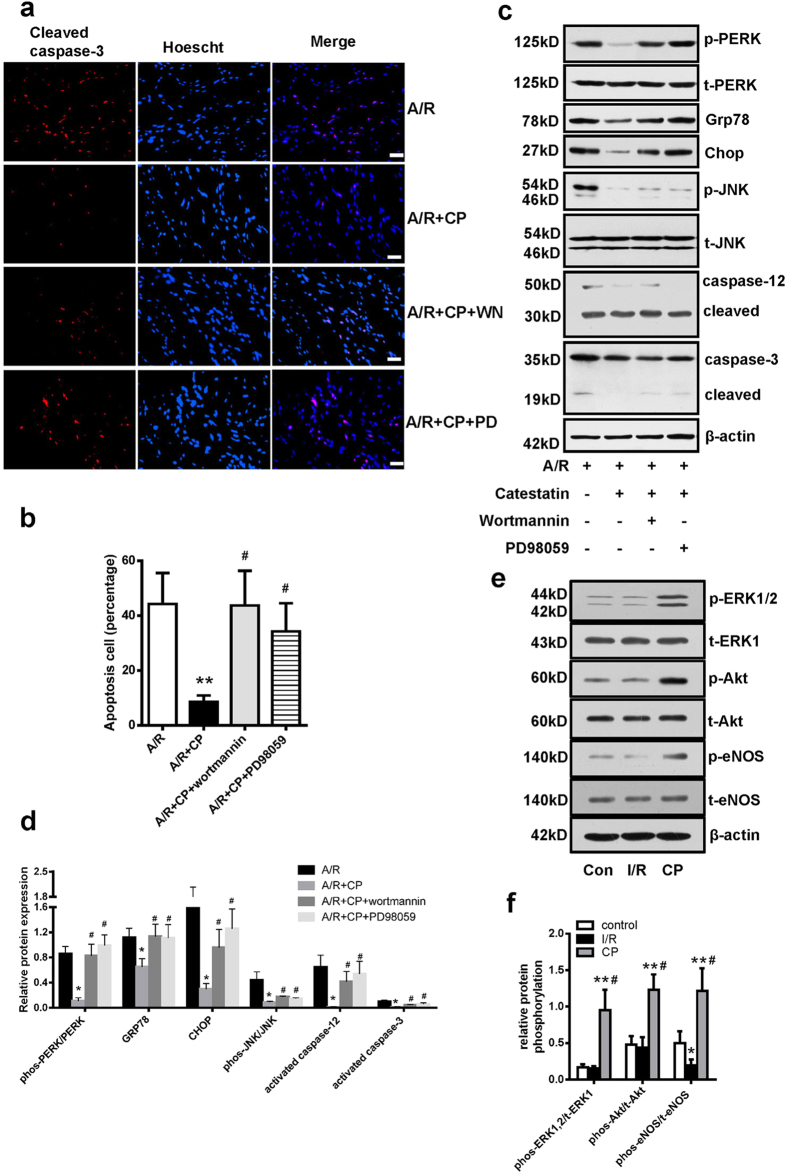
The role of ERK1/2 and PI3 K/Akt pathway in CST’s protective effects during A/R in cardiomyoblasts. H9c2 cardiomyoblasts were pretreated with ERK inhibitor PD98059 (20 μM), PI3 K/Akt inhibitor wortmannin (10 nM) for 30 min, followed by treatment with CST (100 nM) for 30 min, then cells underwent A/R. (**a**) Apoptosis was assessed by cleaved caspase-3–positive cells (**a**) and counted (**b**). Scale bars = 50 μM. Western blot analysis of ER stress-markers expression(**c**), and relative protein expression was analyzed (**d**). Six independent experiments were performed for above studies. (**e**) Western blot analyzed the changes of phosphorylated ERK1/2, Akt and eNOS during CP in isolated heart; and expressions analysis (**f**) was showed. Three independent experiments were done for it.

To investigate the possible signals of CST protection, we treated H9c2 cells with specific inhibitors for 30 min to assess the blocking effects. Intriguingly, PD98059 and wortmannin reversed the CST protection on cell apoptosis (Fig. 4a,b) and ER-stress response (Fig. 4c,d). Knockdown CST also lowered the phosphorylation of ERK1/2 and Akt during A/R (see supplementary Fig. S6 online). These results indicate that ERK1/2 and PI3 K pathway involved in the protection of CST. In isolated heart, we also found CP significantly increased ERK1/2, Akt and eNOS phosphorylation (Fig. 4e,f) compared with I/R, which confirmed ERK1/2 and PI3 K/Akt were partly activated by CST to reduce the ER-stress and increase cell survival.

**Figure 5:**
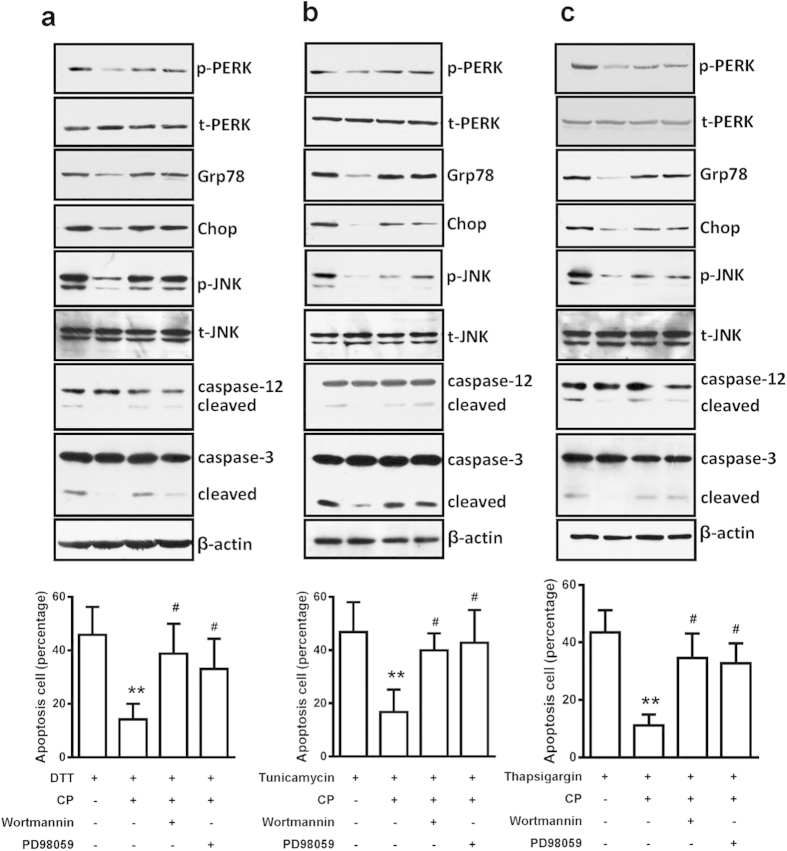
CST protected against ER-stress induced apoptosis via ERK1/2 and PI3 K pathway in H9c2 cardiomyoblast. Western blot analysis of protein expression of ER stress-marker proteins and apoptosis cell number with treatment with stress inducers dithiothreitol (DTT) (**a**), tunicamycin (**b**), and thapsigargin (**c**) after pretreatment with PD98059 or wortmannin. **P < 0.01 vs single ER-stress inducers, ^#^P < 0.05 vs single catestatin treatment. Eight independent experiments were performed for above studies.

To confirm these pathways involved in ER stress-induced cellular apoptosis by CST, we measured the effect of these inhibitors on DTT-, tunicamycin- and thapsigargin-induced cellular apoptosis. *In situ* staining of apoptotic cells with cleaved caspase-3 antibody revealed that CST reduced the number of apoptotic cells induced by DTT, tunicamycin and thapsigargin, and which also were blocked by PD98059 and wortmannin (see supplementary Fig. S7–S9 online). Consistently, the major apoptosis pathway of ER stress—Chop expression, caspase-12 cleavage and JNK phosphorylation, ER stress response markers including phosphorylated PERK, Grp78 protein expression—were also lowered by CP while inducing by DTT (Fig. 5a, and supplementary Fig. S10 online), tunicamycin (Fig. 5b and supplementary Fig. S11 online) and thapsigargin (Fig. 5c and supplementary Fig. S12 online), which were also reversed by two inhibitors. Therefore, CST inhibited apoptotic pathways of the unfolded protein response in part via ERK and PI3 K signaling pathways.

### CST acts as an M2 receptor agonist

**Figure 6:**
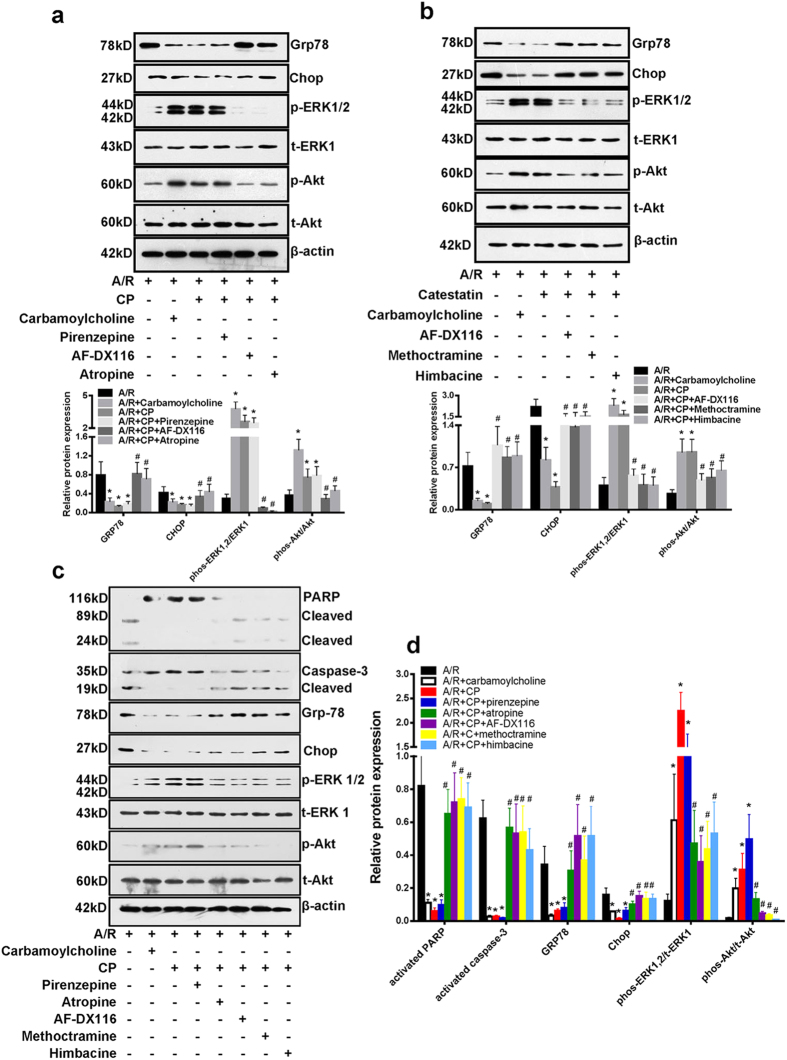
The role of the muscarinic receptors in CST’s protective effect on apoptosis. (**a**) Western blot analysis of effect of non-selective M receptor antagonist atropine, selective M1 receptor antagonist pirenzepine and selective M2 receptor antagonist AF-DX116 on ER-stress markers expression and phosphorylation of ERK1/2 and Akt and (**b**) M2 receptor-specific blocking confirmed with methoctramine and himbacine. Quantitative analysis of protein expression was shown in the bottom of figure. *P < 0.05 vs A/R treatment; ^#^P < 0.01 vs A/R + CP group. Six independent experiments were performed for above studies. In neonatal rat cardiomyocytes, we measured the apoptosis markers-PARP and caspase-3 cleavage, ER-stress markers-Grp78 and Chop expression, ERK1/2 and Akt phosphorylation; and the role of M2 receptor using selective M2 receptor antagonists(**c**). Relative protein expression was performed (**d**). Three independent experiments were performed for it.

CST might activate G_i/o_ protein^20^. In 5 subtypes (M1 to M5) muscarinic acetylcholine receptors, only M_2_ and M_4_ receptors bind with G_i/o_ protein^21^. Thus, muscarinic acetylcholine receptor signaling might be involved in the cardioprotective effect of CST. To verify this hypothesis, the nonspecific M receptor antagonist atropine, selective M_1_ receptor antagonist pirenzepine, and selective M_2_ receptor antagonist AF-DX116 were used. We found that carbamoylcholine lowered Grp78 and Chop protein expression and increased phosphorylated ERK1/2 and Akt levels effect which was similar to the CST (Fig. 6a). Atropine and AF-DX116 but not pirenzepine blocked the CST-altered level of these proteins (Fig. 6a). Therefore, M_2_ but not M_1_ receptor may mediate the CST action. To confirm this finding, we used another selective M_2_ receptor antagonist-methoctramine, and the selective M_2_/M_4_ receptor antagonist himbacine, which also blocked the effect of CST on the ER stress response and ERK1/2 and Akt phosphorylation during A/R (Fig. 6b). In isolated neonatal rat cardiomyocytes, CP also reduced cell apoptosis (PARP and caspase-3 cleavage), ER-stress response (Grp78 and Chop protein expression) by I/R, and increased phosphorylated ERK1/2 and Akt. The protection were also blocked with selective M2 receptor inhibitors (Fig. 6c,d). In isolated heart, AF-DX116 also blocked the protective effects of CP on infarct size, LDH leakage and cTNI level (supplementary Fig. S13a online). These data suggested that M_2_ receptor may be a target protein interacting with CST.

**Figure 7:**
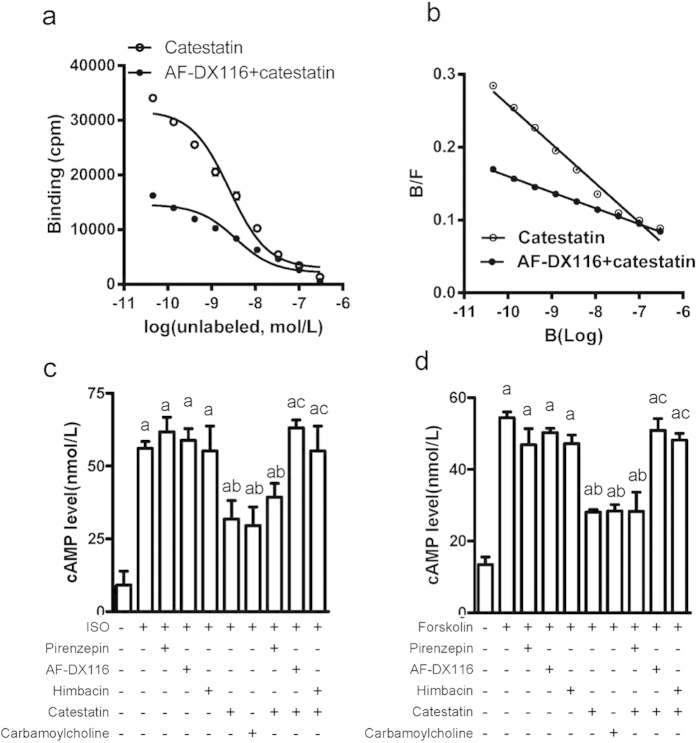
CST bound to M2 receptor and reduced cellular cAMP levels. (**a**) Competitive inhibitory receptor binding curve involved [^125^-I]-labeled CST (0.4 μM) as a ligand, N-methylscopolamine (NMS, 0.045 nM to 0.3 μM) as “cold” ligand, and AF-DX116 as selective inhibitor. (**b**) Equilibrium dissociation constant (*KD*) values were counted by scatchard analysis. Selective M-receptor antagonist effect with CST on intracellular cAMP level after stimulation with isoproterenol (**c**) or forskolin (**d**). Data are mean ± SD. (**a**) P < 0.05 vs untreated group; (**b**) P < 0.01 vs single treatment with isoproterenol (ISO) or drugs; (**c**) P < 0.01 vs CST. Six independent experiments were performed for these studies.

To investigate the interaction of CST with M_2_ receptor and as an endogenous ligand, we used radioactive isotope [^125^-I]-labeled CST (0.4 μM) as a ligand, and N-methylscopolamine (NMS:0.045 nM to 0.3 μM) as a “cold” ligand were co-incubated with membrane protein (200 μg) in H9c2 cardiomyblasts. Radioactivity of binding CST was assessed and the competitive inhibition curve is in Fig. 7a. The half-maximal inhibition (IC_50_) of binding occurred with 25.7 nM NMS (Fig. 7a). Scatchard analysis revealed a single binding site with *KD* 18.6 nM (Fig. 7b). To confirm the specific binding of CST to the M_2_ receptor, we added AF-DX116 (final concentration: 50 nM, IC_50_ 50 nM for M_2_ receptors), and the IC_50_ of binding was significantly increased to 36.0 nM (Fig. 7a); scatchard analysis revealed a *KD* of 45.8 nM (Fig. 7b). Therefore, CST specifically binds to the M2 receptor.

Activation M2 receptor can decrease intracellular cAMP level under catecholamine stimulation. To identify the biological function of CST binding to the M2 receptor, we measured cAMP level with carbamoylcholine as a positive control. CST and carbamoylcholine lowered cAMP level induced by isoproterenol in H9c2 cells (Fig. 7c). AF-DX116 and himbacine did not affect increased cAMP level with isoproterenol, but blocked the effect of CST. To distinguish the β-receptor inhibition, we used the adenylyl-cyclase activator forskolin to directly increase intracellular cAMP level. CST also lowered cAMP level, which was blocked by AF-DX116 and himbacine but not pirenzepin (Fig. 7d). Thus, CST functions as an agonist of the M2 receptor.

## Discussion

**Figure 8:**
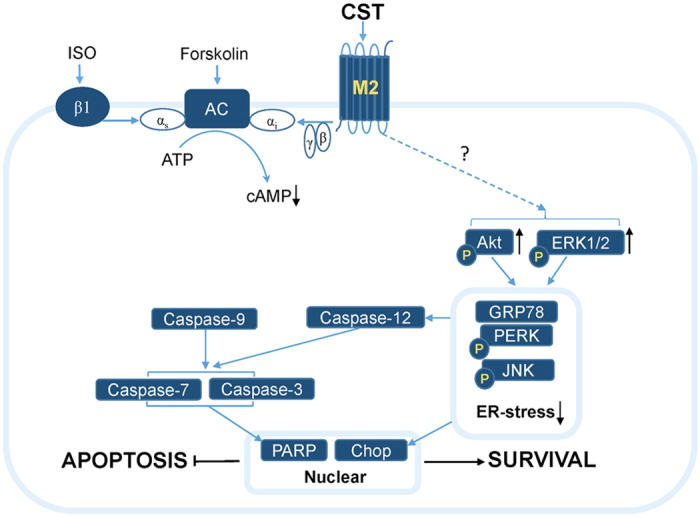
Proposed mechanism of CST’s protective effects in cardiac I/R injury. CST acts as an endogenous M2 receptor agonist, activates M2 receptor and causes inhibition of adenylyl cyclase (AC) activity via the α subunit (αi) of Gi, thereby decreasing cAMP level while stimulating by isoproterenol or forskolin. During cardiac I/R, CST binds to M2 receptor and activates ERK1/2 and PI3 K/Akt signaling pathway to reduce ER-stress response and cell apoptosis.

In the present study, we identify the CST is an endogenous ligand of the M2 receptor. CST pretreatment activates M2 receptor, thereby activates PI3 K and ERK1/2 pathway results in reducing ER stress-induced cell apoptosis and increasing cell survival (Fig. 8), thus lowers infarct size and recovers cardiac pump function under I/R.

Firstly we identified the endogenous CST reducing after I/R, but its precursor ChgA expression were no changes. That means increased enzymes (which catalyzed ChgA to generate CST) activity during I/R, although the enzymes were not identified until now. CST pretreatment compensated to endogenous CST decrease, thus lowered the infarct size and improved cardiac function in rats under I/R, which agreed with studies of CST post-conditioning^16^,^17^. However, human wild type CST (100 nM) treatment for 2 h during reperfusion phase increased, but Gly^364^Ser variant peptide lowered infarct size; because of lower inhibitory activity on nicotinic cholinergic receptor^22^. In our present study or CST post-conditioning, short-times supplementation reduced the inhibitory effects on nicotinic cholinergic receptor, which might partly explain the paradoxical effects of CST on cardiac I/R.

Apoptosis occurs in cardiomyocytes during I/R. Genetic or pharmacological inhibition of cell apoptosis reduced infarct size and improved cardiac function^23^. CST pretreatment similar with CST post-conditioning^24^, decreased apoptosis during I/R. In contrast, knockdown CST increased apoptosis. ER stress induces apoptosis involved in regulation during I/R^25^. CST pretreatment lowered ER stress induced apoptosis during I/R, even directly stimulated by ER-stress inducers; oppositely, knockdown CST aggravated ER-stress induced apoptosis, suggesting endogenous CST stabilized ER-stress response to anti-apoptosis.

Next, we assessed the possible signal involved in the CST regulation. A PI3 K inhibitor blocked the Ca^2+^ transient inhibition of CST after β-adrenergic stimulation^11^ and cardiac function recovery with CST post-conditioning^17^. We found that wortmannin blocked the effect of CST on ER stress-linked apoptosis; and knockdown CST lowered phosphorylated Akt. Therefore, the PI3 K-Akt pathway is an important signal cascade of CST in cardiac diseases. The canonical mitogen-activated protein kinases (MAPKs) JNK, ERK1/2 and p38 are activated in response to ER stress, each with different roles. JNK is considered a downstream target of IRE1-dependent ER stress^26^. I/R–induced ROS leads to sustained calmodulin-dependent protein kinase II activation, which promotes JNK signaling^27^, thereby promoting apoptosis^26^. Here we found that CST inhibited JNK phosphorylation, and siRNA knockdown of CST activated JNK. Thus, the JNK signal is involved in inhibiting ER stress-induced apoptosis with CST. In general, ERK1/2 activation is considered to promote cell survival. ER stress-promoted activation of ERK1/2 is associated with increased cell proliferation and decreased apoptosis^28^. Pharmacological or genetic inhibition of ERK1/2 promoted ER stress-induced apoptosis^29^. We found that PD98059-inhibited MAPK kinase reduced ERK1/2 activation and reversed the protection of CST on ER stress, so CST increase ERK1/2 phosphorylation to promote cell survival. These findings indicate that PI3 K and ERK1/2 pathway involved in the protection of endogenous CST during I/R.

We asked how CST activates PI3 K and ERK1/2 signal and which the receptor of CST is. Here we showed CST was similar to carbamoylcholine, reduced ER-stress response and activated ERK1/2 and PI3 K signals. This effect was blocked by a selective M2 but not M1 receptor antagonist. Receptor binding assay showed that CST is a ligand of the M2 receptor. CST bound to M2 receptor activation directly activating G_i_ protein, thus reducing cAMP production while stimulated by isoproterenol^30^ or forskolin; and the effect was also blocked by M2 selective antagonism, suggesting CST functions as an M2 receptor agonist. M2 receptor activation increases the NOS/NO cGMP pathway^31^, which also explains in part the CST anti-adrenergic effect by NOS/NO cGMP^11^. All these findings demonstrate that endogenous CST as an agonist of M2 receptor to participate in pathogenesis of the cardiac I/R.

Muscarinic receptor expression is abundant in ventricular muscle. Acetylcholine has protective role in cardiac I/R *in vitro*, but it is non-selective ligand and activates all cholinergic receptor subtype, which limited its application due to enormous side effects. Here we identified that CST is a selective ligand of M2 receptor with high affinity and its intracellular signal (cAMP, PI3 K and ERK1/2) is consistent with M2 receptor agonist. Berberine also has potential binding ability to M2 receptor (*KD* values 5.4 μM) and has protective effects in cardiac diseases^32^. These data indicate that M2 receptor might be a new target for cardiac I/R, but it should be confirmed in cardiomyocytes specific M2 receptor knockout mice in the future. Different amino acid variant in CST affected CST inhibitory activity on nicotinic cholinergic receptor^33^. Thus, we also increasing CST binding to M2 receptor by modification replacement amino acid, if the sites and model of CST binding to M2 receptor are clear. A synthesized small-molecule chemical according to the pharmacophore model of CST lowered blood pressure in hypertensive animals^34^. According to the model, more selective and effective M2 agonist might be designed and synthesized for treating cardiac ischemic diseases.

## Methods

### Animals and materials

All animal procedures complied with the Animal Management Rule of the Ministry of Health, People’s Republic of China (document No. 55, 2001) and the Care and Use of Laboratory Animals published by the US National Institutes of Health (NIH Publication No. 85-23, updated 2011). The care and use of the laboratory animals were approved by Laboratory Animal Ethics Committee of Peking University. Total 36 male Sprague–Dawley (SD) rats (180–200 g, 8 week) were supplied by the Animal Center, Health Science Center, Peking University. H9c2 rat cardiomyoblasts were purchased from the Stem Cell Bank, Chinese Academy of Sciences. The human wild-type CST, hChgA_352–372_ (SSMKLSFRARGYGFRGPGPQL), was purchased from Phoenix Pharmaceuticals (Belmont, CA, USA). The extracellular signal-regulated kinase (ERK) inhibitor PD98059, PI3 K/Akt inhibitor wortmannin, selective M_1_ receptor antagonist pirenzepine, selective M_2_ receptor antagonists AF-DX116 (Otenzepad) and methoctramine, selective M_2_/M_4_ receptor antagonist himbacine, and non-specific M receptor antagonist atropine, carbamoylcholine, tunicamycin, dithiothreitol (DTT), and thapsigargin were from Sigma-Aldrich (St. Louis, MO, USA). ChgA siRNA (sense: GAU GAU GAU GGU CAG UCG GdTdT, antisense: CCG ACU GAC CAU CAU CAU CdTdT) and scramble siRNA were synthesized by Sigma.

### Langendorff perfusion procedure and experimental protocol

Rats fed a standard diet were heparinized (200 IU) and anesthetized with sodium pentobarbital (50 mg/kg, intraperitoneally). Hearts were excised and rapidly moved to a Langendorff perfusion system as described^35^. After 30-min stabilization, hearts underwent 30-min normoxia, zero-flow global ischemia for 30 min, followed by 60-min reperfusion (I/R group). For the CP group, CST (100 nM) was infused for 15 min before ischemia (Fig. 1A). Cardiac function including left ventricular end diastolic pressure (LVEDP), LV systolic pressure (LVSP), and maximal and minimal LV pressure development (±dp/dt_max_) were recorded.

At the end of reperfusion, hearts were frozen at −20 °C, then cut into 2-mm thin slices, which were stained with 1% triphenyltetrazolium chloride (TTC) in phosphate buffer (pH 7.4) at 37 °C for 10 min, then fixed in 4% paraformaldehyde solution to enhance the contrast of the stain. Images in each slice were analyzed by image pro plus software. The volume of infarcted tissue (white) and the risk zone (total) was then calculated by each area plus slice thickness and summing the results. The infarct size was expressed as the percentage of the risk zone^36^. Total 12 rats were sacrificed for the infarct size assay. Other rats, while finished I/R procedure, the apex of heart (about 100 mg) was quickly removed for western blot assay. The residual heart were cut about 5 mm high (from the site of apex), embedded with OCT then the frozen slice were prepared for apoptosis staining (Methods of western blot and apoptosis assay are available in the Supplementary methods online).

### Anoxia/reoxygenation (A/R) and induced ER stress in H9c2 and neonatal cardiomyocytes

Ten two-day-old Sprague–Dawley rats were anesthetized with sodium pentobarbital (50 mg/kg, intraperitoneally), and then decapitated by heavy scissors. Their ventricles were excised and dissociated using trypsin digestion as described previously^37^. The isolated cells were pre-plated for 0.5 h in minimal essential medium with 10% fetal calf serum. Nonattached cells were re-plated in another plate in the same medium with 0.1 mM bromodeoxyuridine (BrDU) for 4 days and then were removed. The neonatal cardiomyocytes were used for experiments after a further 2 or 3 days.

H9c2 rat cardiomyoblasts or neonatal cardiomyocytes were cultured in DMEM with 10% fetal bovine serum (FBS) and 1% penicillin/streptomycin at 37 °C, 5% CO_2_. Medium was changed to fresh medium (serum-free), and cells were cultured under anoxia with 95% N_2_ and 5% CO_2_ for 12 h at 37 °C, then incubated in a standard incubator for 4 h (A/R)^38^.

Cells were pretreated with PD98059 (20 μM), wortmannin (10 nM), atropine (10 nM), pirenzepine (1 μM), AF-DX116 (100 nM), methoctramine (10 nM), or himbacine (1 μM) for 30 min, then CST (100 nM) or carbamoylcholine (100 μM) for 30 min, then underwent A/R or were treated with the ER stress inducers tunicamycin (5 μg/mL, 8 h), dithiothreitol (5 mM, 4 h), or thapsigargin (5 μM, 8 h).

Cells at 40% to 50% confluence were transfected with siRNA or scramble siRNA (50 pM) by use of Lipofectamine 2000 (Invitrogen), then incubated at 37 °C in a CO_2_ incubator for 48 h before A/R.

### Competitive receptor binding assay

Membranes were prepared from H9c2 cells as described^39^. Membrane protein was resuspended in HEPES buffer (20 mM HEPES, 1 mM MgCl_2_, pH 7.4). An amount of 200 μg membrane protein was incubated in 1 mL HEPES buffer containing 0.4 μM [^125^I]-labeled CST (200000 cpm) for 60 min at 37 °C. [^125^I]-labeled CST dissociation was initiated by the addition of N-methylscopolamine (NMS, 0.045 nM to 0.3 μM) with and without AF-DX116 (50 nM). A control with nothing added was included in each experiment. Binding was terminated by rapid vacuum filtration onto Whatman GF/B filters. In all experiments, non-specific binding was determined with 1 μM NMS. Filter-bound radioligand was measured by use of a scintillation counter (PerkinElmer, MA, USA). Data were analyzed by non-linear regression with Prism.

### Statistical analysis

Statistical analysis was performed by SPSS Statistics version 15.0 for windows. Data are presented as mean ± SD. Differences among groups were analyzed by one-way ANOVA, then Student-Newman-Keuls test. Differences between 2 groups were analyzed by two-tail nonparametric *t* test.

## Additional Information

**How to cite this article**: Liao, F. *et al*. Catestatin attenuates endoplasmic reticulum induced cell apoptosis by activation type 2 muscarinic acetylcholine receptor in cardiac ischemia/reperfusion. *Sci. Rep.* **5**, 16590; doi: 10.1038/srep16590 (2015).

## Supplementary Material

Supplementary Information
