# Author Correction: Catestatin attenuates endoplasmic reticulum induced cell apoptosis by activation type 2 muscarinic acetylcholine receptor in cardiac ischemia/reperfusion

**DOI:** 10.1038/s41598-020-76697-9

**Published:** 2020-11-05

**Authors:** Feng Liao, Yang Zheng, Junyan Cai, Jinghui Fan, Jing Wang, Jichun Yang, Qinghua Cui, Guoheng Xu, Chaoshu Tang, Bin Geng

**Affiliations:** 1 Department of Physiology and Pathophysiology, School of Basic Medical Science, Peking University, Beijing, P.R. China; 2 Center for Noncoding RNA Medicine, Peking University Health Science Center, Beijing, 100191 China

Correction to: *Scientific Reports* 10.1038/srep16590, published online 16 November 2015

This Article contains errors.

As a result of a figure assembly error, Figure 3D panel Scrambled is a duplication of Figure 4A panel A/R+CP+PD. The correct Figure 3D appears below as Figure 1.

**Figure 1:**
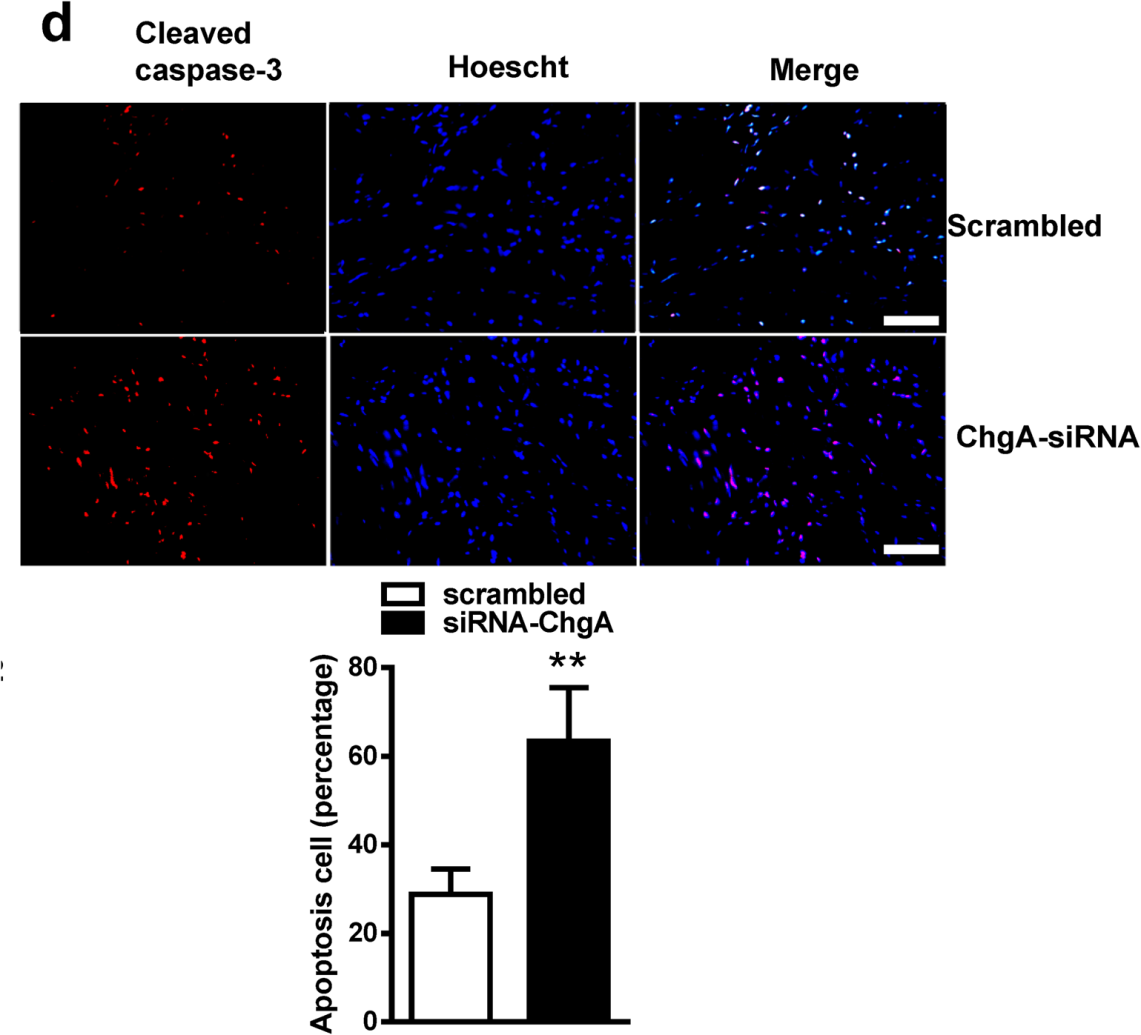
The correct version of Figure 3D.

This change does not affect the conclusions of the Article.

