# Supplementary material for: Catestatin attenuates endoplasmic reticulum induced cell apoptosis by activation type 2 muscarinic acetylcholine receptor in cardiac ischemia/reperfusion
Source: Sci Rep. 2015 Nov 16;5:16590. doi: 10.1038/srep16590 (PMC4645123; doi:10.1038/srep16590)
Supplement: Supplementary Information [file srep16590-s1.pdf]

Catestatin attenuates endoplasmic reticulum induced cell apoptosis by activation type 2 muscarinic acetylcholine receptor in cardiac ischemia/reperfusion

Feng Liao[1]*, Yang Zheng[1]*, Junyan Cai[1], Jinghui Fan[1], Jing Wang[1], Jichun Yang[1,2], Qinghua Cui[1,2], Guoheng Xu[1], Chaoshu Tang[1], Bin Geng[1,2]

1. Department of Physiology and Pathophysiology, School of Basic Medical Science, Peking University, P.R. China.
2. Center for Noncoding RNA Medicine, Peking University Health Science Center

Online Methods

1. Western blot analysis

Cultured H9c2 cells or rat hearts were lysed in RIPA lysis buffer (Cell Signaling). Proteins were denatured and 50 μg protein was loaded and isolated by SDS-PAGE electrophoresis, then transferred to a nitrocellulose membrane. After being blocked, membranes were incubated overnight at 4°C with primary antibody for CHOP (1:500, sc-575), phospho-ERK1/2 (1:1000, sc-16981R), ERK1 (1:1000, sc-93), phospho-Akt (1:1000, sc-7985-R), Akt (1:1000, sc-8312), ChgA (1:500, sc-376813), β-actin (1:5000, sc-47778; all Santa Cruz Biotechnology) or apoptosis antibodies (for caspase-3, -7, -9, -12, PARP; 9915S), GRP78 (3183), phospho-PERP (3719), PERP (3192), phospho-Plus SAPK/JNK (Thr183/Tyr185) (8206; all 1:1000, all Cell Signaling). The antigens were detected by the chemiluminescence method (ECL western blot detection kit, Applygen) with horseradish peroxidase-linked anti-mouse or anti-rabbit secondary antibody.

2. Immunofluorescence staining for apoptosis cells

Frozen heart sections (6 μm) were prepared. H9c2 cells were washed with cold phosphate buffered saline (PBS), fixed in 4% formaldehyde for 20 min, then treated with 0.1% Triton X-100. Sections and cells were blocked with 1% bovine serum albumin (BSA) and incubated overnight at 4°C with a primary antibody against cleaved-caspase-3 (1:200, Cell Signaling). After being washed with PBS, sections and cells were incubated for 60 min with DyLight 633-labeled secondary antibody (1:400, Jackson), then incubated with Hoechst 33258 solution (50 ng/mL) for 10 min. Images were captured by fluorescence microscopy and apoptotic cells were analyzed by use of Image-Pro Plus.

3. cAMP Assay

H9c2 cells were subcultured at 1 x 10^4 cells in 96-well plates. Cell were pretreated with

pirenzepine (1 μM), AF-DX116 (100 nM) or himbacine (1 μM) for 20 min, then incubated with CST (50 nM) or carbamoylcholine (100 μM) along with isoproterenol (1 μM) or forskolin (100 μM) for 30 min. Cell underwent cAMP assay with the cAMP-Glo Max Assay Kit (Promega).

Online Figures

Fig.S1

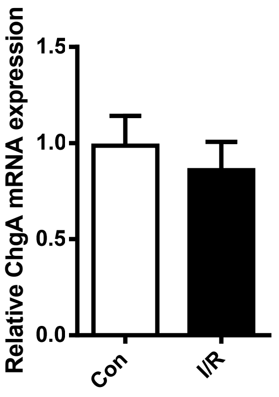

Figure S1. Changes of ChgA mRNA expression after ischemia/reperfusion (I/R). Langendorff-perfused heart underwent 30 min ischemia and then 1 h reperfusion. Total RNA were extracted and RT-quantitative PCR were assayed for ChgA.

Fig.S2

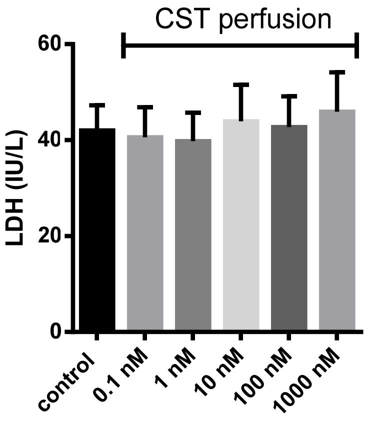

Figure S2. The effects of CST at different concentration on lactate dehydrogenase (LDH) leakage (a marker of heart injury) in langendorff-perfused heart.

Fig.S3

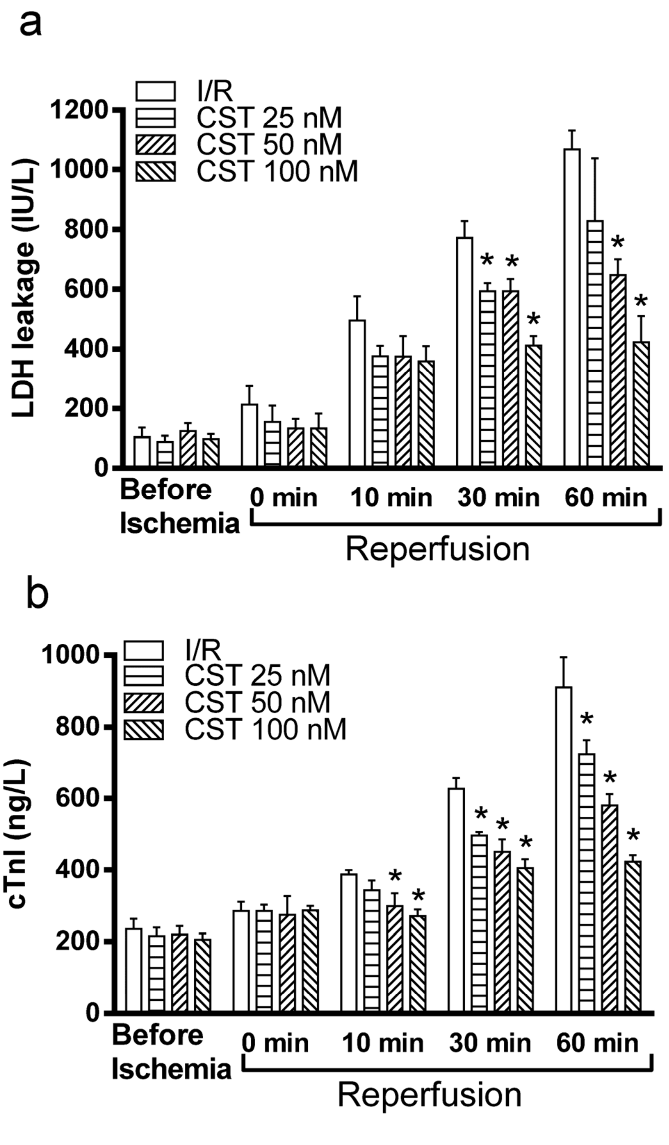

Figure S3. The changes of LDH and cardiac troponin I (cTNI) level in perfusate by different concentration of CST pretreatment. Assessment of LDH leakage (a) or cTNI content (b) in perfusate in before ischemia and reperfusion at 0 min, 10 min, 30 min and 60 min while giving different concentration of CST (25 nM, 50 nM and 100 nM) pretreatment. Data are mean±SD. *P<0.05 vs I/R at corresponding time point.

Fig.S4

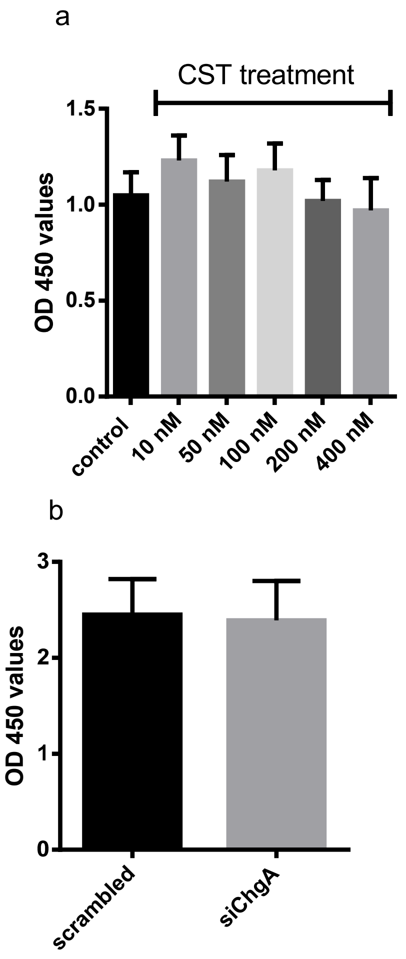

Figure S4. The alterations of cell viability by CST treatment. (a) H9c2 cell were treated with different concentration of CST for 24 h, or siRNA-mediated knockdown CghA for 48 h (b), cell viability were assessed by CCK8 kit.

Fig.S5

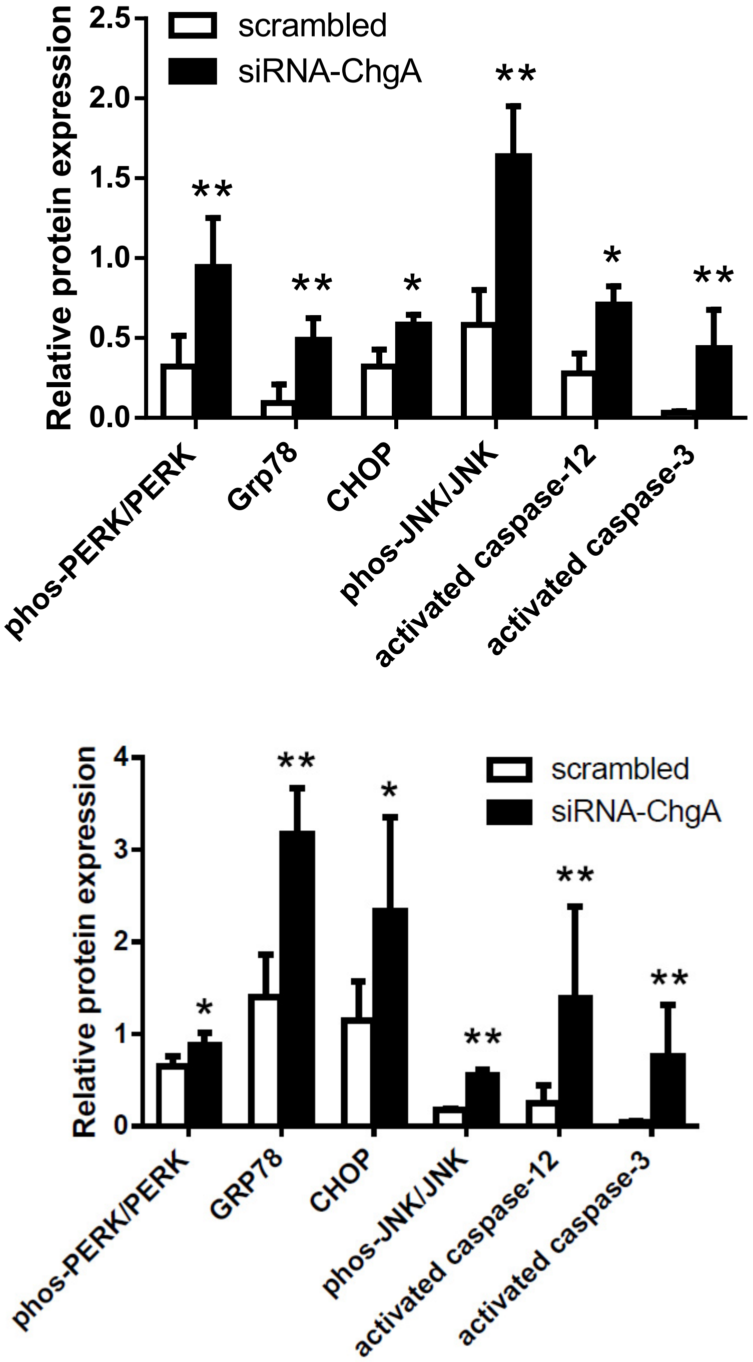

Figure S5. Using siRNA knockdown CST precursor-ChgA. Quantitative analysis of protein expression of H9c2 cardiomyoblast cell underwent A/R (top figure) or thapsigargin (bottom figure). Data are mean±SD. *P<0.05; ** P<0.01 vs scrambled RNA.

Fig.S6

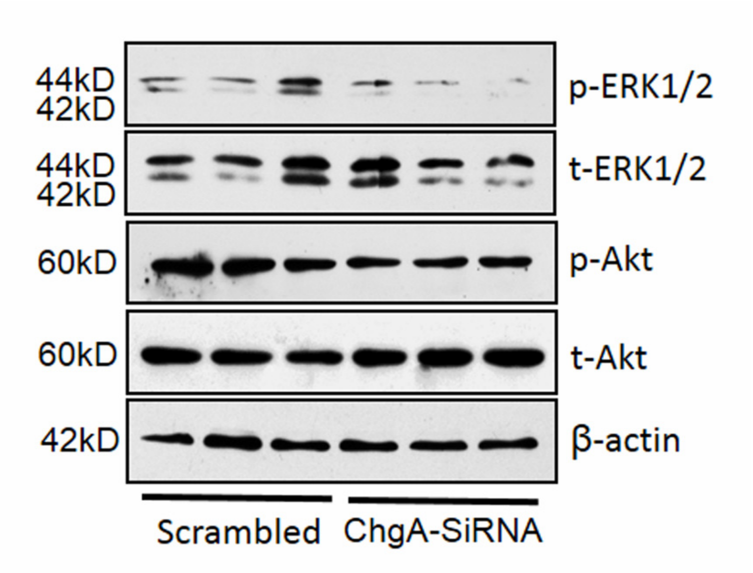

Figure S6. Western blot analysis of phosphorylated and total extracellular signal-regulated kinase 1/2 (ERK1/2) and Akt with siRNA knockdown of the CST precursor ChgA in H9c2 cardiomyoblasts cultured for 2 h under anoxia, then 4 h under normal conditions (A/R).

Fig.S7

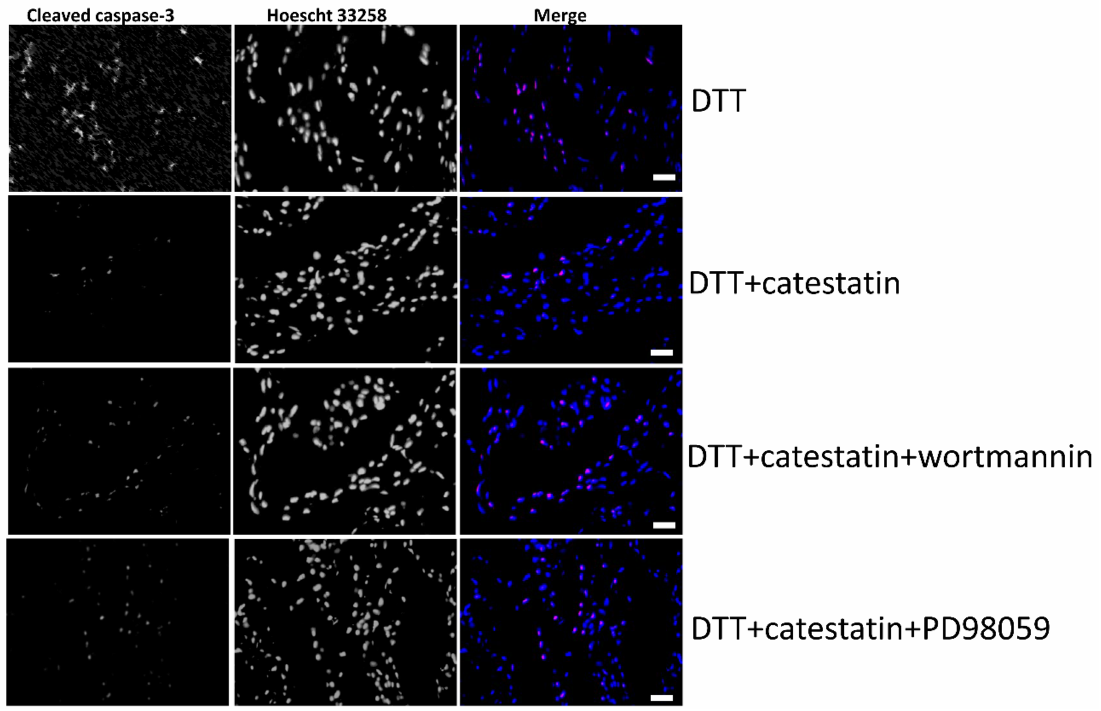

Figure S7. Immunohistochemistry of H9c2 cardiomyoblasts pretreated with wortmannin, PD98059 or H89 for 30 min, then CST for 30 min, then treatment with ER stress inducer DTT for 4 h. Apoptosis is indicated by cleaved caspase-3 staining.

Fig. S8

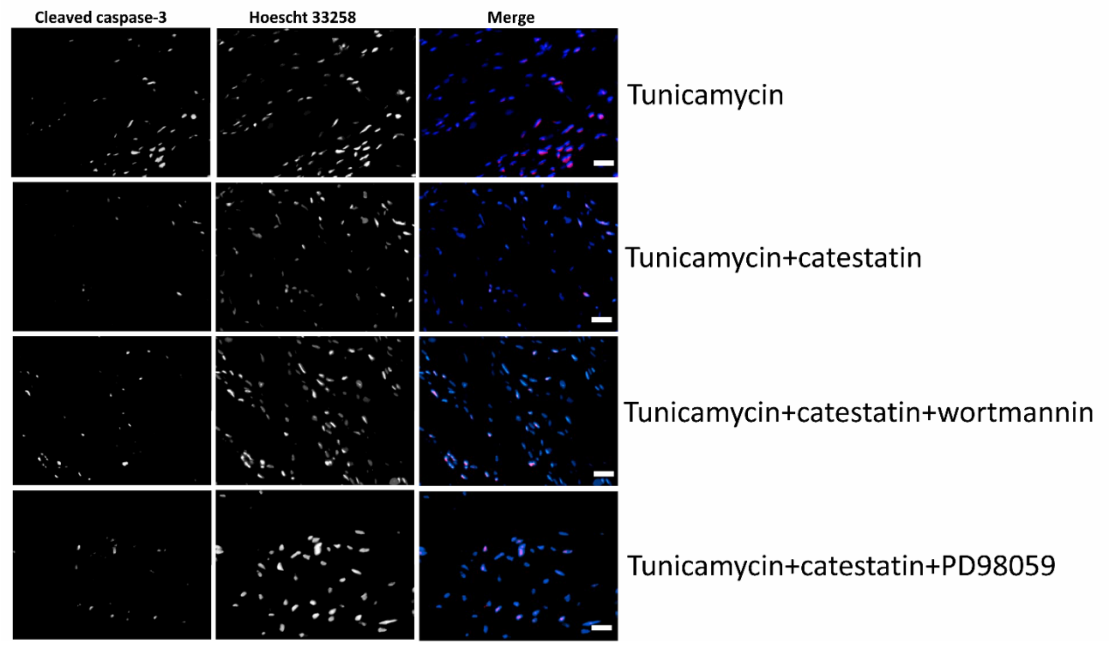

Figure S8. Immunohistochemistry of effect of wortmannin, PD98059 and H89 on CST inhibiting cell apoptosis (cleaved caspase-3–positive cells) with ER stress inducer tunicamycin for 8 h.

Fig.S9

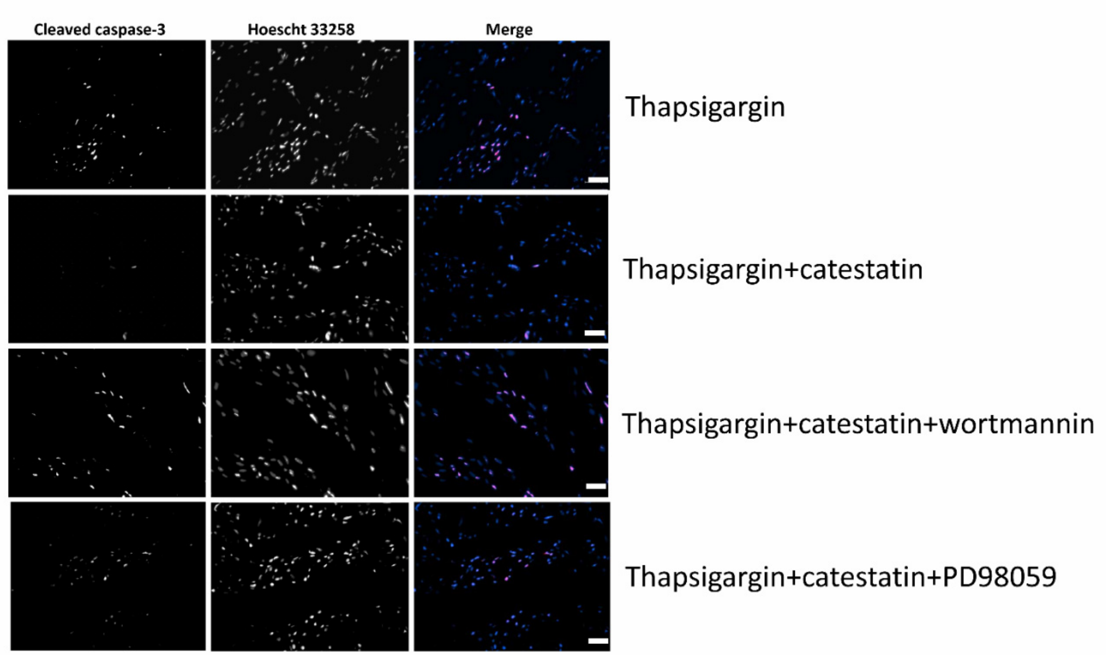

Figure S9. Immunohistochemistry of effect of wortmannin, PD98059 and H89 on CST inhibiting cell apoptosis (cleaved caspase-3–positive cells) with ER stress inducer thapsigargin for 8 h.

Fig.S10

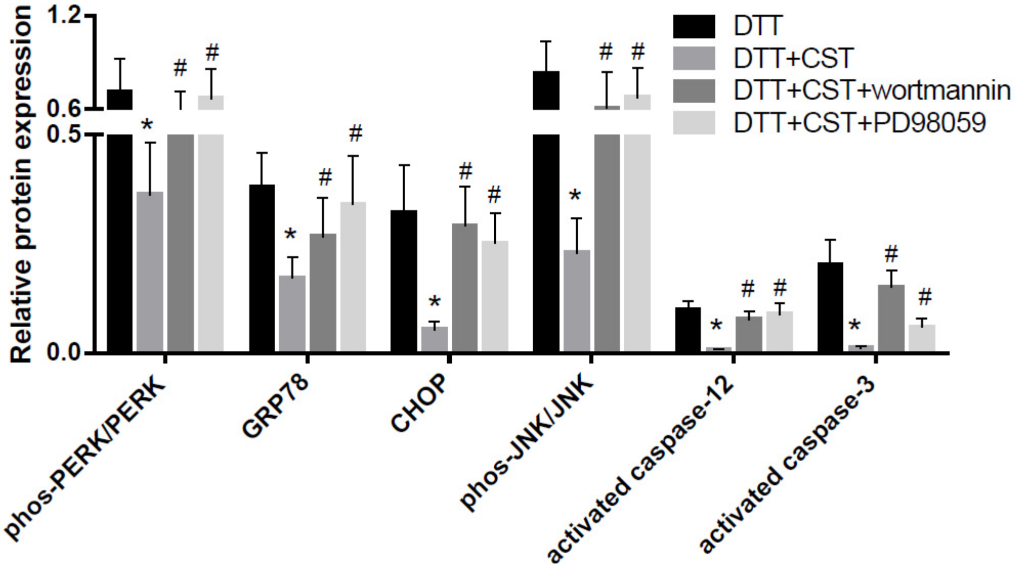

Figure S10. Quantitative analysis of protein expression for Figure 5a. Relative protein expression for activated PARP and caspase-3 means the ratio of cleaved fragment to total protein. Relative protein expression for Grp 78 and Chop means relative protein level to β-actin. Relative protein expression for phosphorylated PERK and JNK means ratio of phosphorylated protein to total target protein. Data are mean±SD. ** $P<0.01$ vs DTT treatment; # $P<0.05$ vs DTT+CST group.

Fig.S11

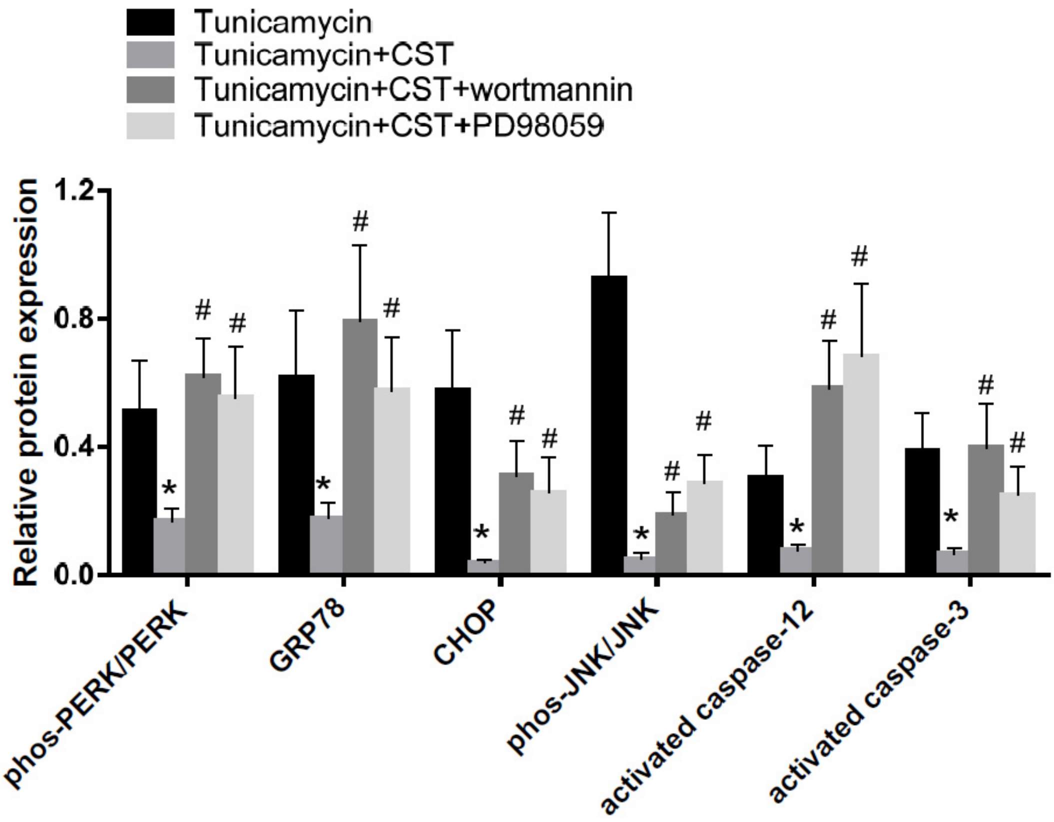

Figure S11. Quantitative analysis of protein expression for Figure 5b. Relative protein expression for activated PARP and caspase-3 means the ratio of cleaved fragment to total protein. Relative protein expression for Grp 78 and Chop means relative protein level to β-actin. Relative protein expression for phosphorylated PERK and JNK means ratio of phosphorylated protein to total target protein. Data are mean±SD. * P<0.01 vs Tunicamycin; # P<0.05 vs Tunicamycin+CST group.

Fig.S12

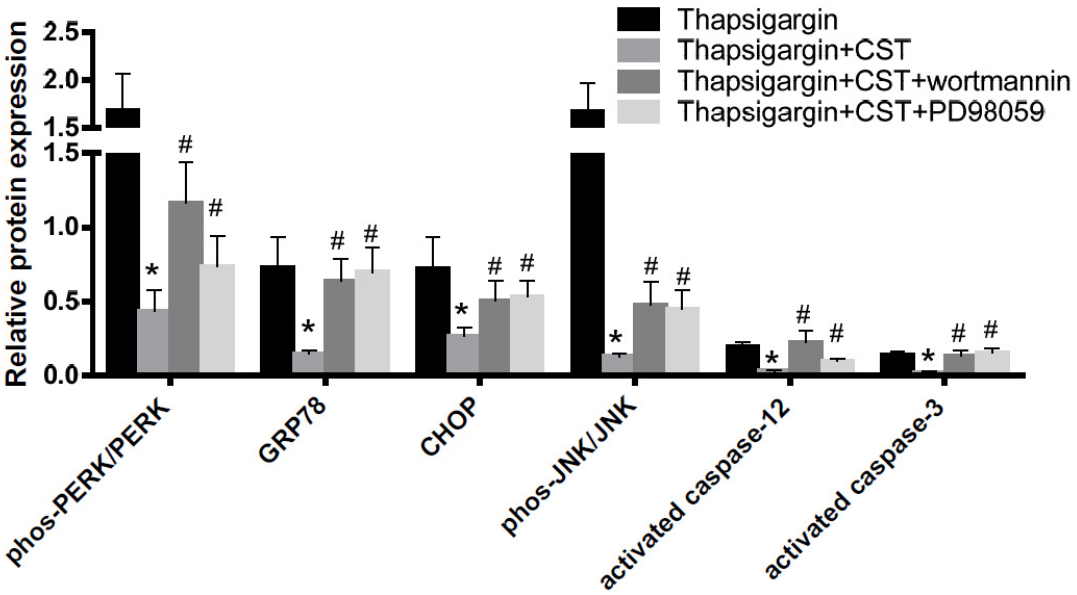

Figure S12. Quantitative analysis of protein expression for Figure 5c. Relative protein expression for activated PARP and caspase-3 means the ratio of cleaved fragment to total protein. Relative protein expression for Grp 78 and Chop means relative protein level to β-actin. Relative protein expression for phosphorylated PERK and JNK means ratio of phosphorylated protein to total target protein. Data are mean±SD. * P<0.01 vs Thapsigargin; # P<0.05 vs Thapsigargin+CST group.

Fig.S13

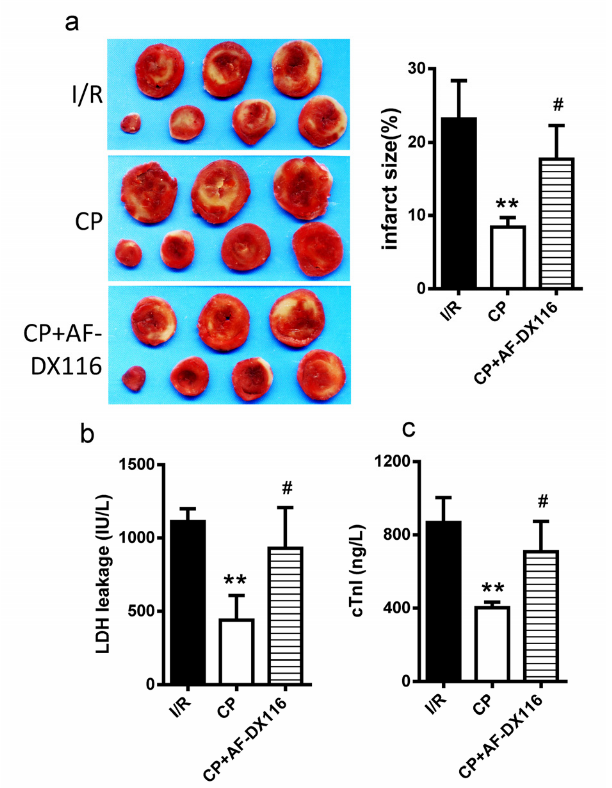

Figure S13: M2 receptor involved in the protection of CP in isolated heart. (a) M2 selective inhibitor-AF-DX116 were pretreating for 20 min before CP, in turn for I/R program, then infarct size were assayed by triphenyltetrazolium chloride stain (left image) and infarct size (white color) was measured by volume (right image). At end of reperfusion, lactate dehydrogenase (LDH) (b) and cardiac troponin I (cTNI) (c) were assayed. Five independent experiments were performed for it. Data are mean ±SD. ** P<0.01 vs I/R group; # vs P<0.01 vs CP group.
